# Factors influencing the need for recovery in employees with hearing loss: a cross-sectional study of health administrative data

**DOI:** 10.1007/s00420-020-01556-z

**Published:** 2020-06-07

**Authors:** Hanneke E. M. van der Hoek-Snieders, Monique Boymans, Bas Sorgdrager, Wouter A. Dreschler

**Affiliations:** 1 Amsterdam UMC, Department of Clinical and Experimental Audiology, University of Amsterdam, Meibergdreef 9, 1105 AZ Amsterdam, The Netherlands; 2 Amsterdam UMC, University of Amsterdam, Netherlands Centre of Occupational Diseases, Coronel Institute AmCOGG, Meibergdreef 9, Amsterdam, The Netherlands

**Keywords:** Workers, Fatigue, Hearing loss, Listening effort, Work participation, Communication strategies

## Abstract

**Objective:**

Need for recovery is a predictor of work stress and health problems, but its underlying factors are not yet well understood. We aimed to identify hearing-related, work-related, and personal factors influencing need for recovery in hearing-impaired employees.

**Methods:**

We retrospectively identified hearing-impaired employees (*N* = 294) that were referred to the Amsterdam University Medical Center between 2004 and 2019. Routinely obtained healthcare data were used, including a survey and hearing assessments. A directed acyclic graph was constructed, revealing the hypothesized structure of factors influencing need for recovery as well as the minimal set of factors needed for multiple regression analysis.

**Results:**

Four variables were included in the regression analysis. In total, 46.1% of the variance in need for recovery was explained by the factors feeling that something should change at work (*B* = 19.01, *p* < 0.001), self-perceived listening effort (*B* = 1.84, *p* < 0.001), personal adaptations scale score (*B* = − 0.34, *p* < .001), and having a moderate/poor general health condition (*B* = 20.06, *p* < 0.001). Although degree of hearing loss was associated with self-perceived listening effort, the direct association between degree of hearing loss and need for recovery was not significant.

**Conclusions:**

The results suggest that the way employees perceive their hearing loss and how they cope with it directly influence need for recovery, rather than their measured degree of hearing loss. Additionally, general health condition was found to be an independent factor for need for recovery. The results should be confirmed by future, longitudinal research.

## Introduction

Hearing loss is a common occupational malady (Backenroth-Ohsako et al. 2003; May 2000). Prevalence estimates vary from 7 to 31% and increase with age and exposure to noise (Hasson et al. 2010; Masterson et al. 2016; May 2000; Nelson et al. 2005). It is estimated that 3% of the Dutch work force experiences difficulties in their job due to their hearing loss (Sorgdrager 2015). These difficulties often result in greater levels of fatigue, fear, social isolation, and psychophysiological stress, caused by the fact that hearing loss goes along with increased listening effort during activities, such as communicating in background noise or localizing sounds (Hornsby and Kipp 2016; Kramer et al. 2006; Morata et al. 2005; Ohlenforst et al. 2017; Svinndal et al. 2018). Adverse implications for work are sick leave due to mental distress, unemployment, and earlier retirement (Danermark and Gellerstedt 2004; Hasson et al. 2011; Kramer et al. 2006; Punch 2016). The degree of hearing loss is significantly associated with need for recovery (NFR) (Nachtegaal et al. 2009), a measure that can contribute to early identification of occupational diseases (de Croon et al. 2003; Moriguchi et al. 2010; Sluiter et al. 2003; Sluiter 1999).

NFR has been defined as the need to recuperate from work induced fatigue, primarily experienced after a day of work (Jansen et al. 2002; van Veldhoven and Broersen 2003). The degree of NFR is determined by the intensity of mental and physical work-induced fatigue and by the period required to return to a normal level of functioning. NFR can be measured with the validated Questionnaire on the Experience and Evaluation of Work (QEEW), which includes 11 dichotomous statements, such as ‘I find it hard to relax at the end of a working day’ and ‘When I get home, people should leave me alone for some time’ (van Veldhoven and Broersen 2003). NFR is a predictor of work stress, subjective health problems, and sick leave (de Croon et al. 2003; Sluiter et al. 2003). In line with the International Classification of Functioning, Disability and Health (ICF) (World Health Organization, 2001). NFR has been described to be a complex construct that is influenced by disease specific, personal, and environmental factors (Gommans et al. 2015).

Despite the importance of the outcome NFR both from health and economic perspectives, the studies examining NFR in patients with hearing loss are scarce. To our knowledge, three studies have been reported so far. In the cross-sectional study by Nachtegaal et al. (2009), the relationship between NFR and hearing status was examined in 925 normally hearing and hearing-impaired working adults. NFR was assessed with the QEEW and hearing status with the national hearing test (Smits et al. 2006), a speech-in-noise test that was performed over the internet. Their regression analysis showed that poorer hearing was significantly associated with higher need for recovery. In the cross-sectional study by Juul Jensen et al. (2018), the relationship between NFR and tinnitus was examined in 32 hearing aid users of which 16 were suffering from tinnitus. NFR was assessed with a Danish translation of the QEEW and tinnitus with the Tinnitus Handicap Inventory. The authors reported that the degree of tinnitus severity was significantly associated with higher NFR. Finally, a randomized controlled trial has been reported by Gussenhoven et al. (2017) in a population of 136 hearing-impaired employees. The study evaluated the effectiveness of a vocational enablement protocol on NFR as compared to usual care for hearing-impaired employees. This protocol is a multidisciplinary program of care that consists of vocational and audiological components, such as an intake interview conducted by the psychologist or social worker and clinical occupational physician, the performance of tone audiometry and a speech-in-noise test, and a multidisciplinary team meeting in which the technical, speech therapeutic, and psychosocial intervention options are discussed (Gussenhoven et al. 2012). The intervention of the control group consisted of any kind of another audiological revalidation. NFR had not significantly changed after 12 months follow-up, and there were no significant differences between the intervention and the control group (Gussenhoven et al. 2017). The authors concluded that NFR may not adequately capture what is covered in the vocational enablement protocol. However, it is unclear how many employees received technical, speech therapeutic, and psychosocial interventions and thus which interventions did not influence NFR. Further, because the factors influencing NFR in hearing-impaired employees are not yet well understood, it is difficult to indicate which changes in degree of hearing loss could have an effect on NFR.

Multiple studies have indicated work characteristics influencing NFR, such as the number of working hours (Jansen et al. 2002; Verdonk et al. 2010), lack of participation in work decisions (van Veldhoven and Broersen 2003), and problems in the relationship with colleagues (Kiss et al. 2008; van Veldhoven and Broersen 2003). High job demands and low job support are associated with high NFR and mixed results are presented for job control (Kiss et al. 2008; Kraaijeveld et al. 2014; Sluiter et al. 2001; Sonnentag and Zijlstra 2006; Van der Hulst et al. 2006). Job demands and job control have also been demonstrated to be associated with NFR in hearing-impaired employees, independently of the degree of hearing loss measured with an online hearing test (Nachtegaal et al. 2009). To our knowledge, further studies examining the effect of work characteristics on NFR in hearing-impaired employees are lacking, but high auditory job demands were shown to be related to sick leave due to stress-related complaints (Kramer et al. 2006).

Personal characteristics influencing NFR in the general working population include gender (Kiss et al. 2008), age (Gommans et al. 2015; Kiss et al. 2008), general health condition (Gommans et al. 2015; van der Starre et al. 2013), educational level (de Croon et al. 2003), and coping style (de Vries et al. 2015; Machin and Hoare 2008). Several studies have indicated that people with hearing loss use coping strategies in their interaction with others (Backenroth-Ohsako et al. 2003; Barker et al. 2017; Hallberg and Carlsson 1991). Also, the Communication Profile for the Hearing Impaired (CPHI) has been developed to investigate how people cope with their hearing loss (Mokkink et al. 2009). This questionnaire contains questions on the communication strategies and non-verbal strategies that are commonly used by people with hearing loss. However, the influence of hearing loss coping on NFR has not yet been examined.

The evidence on factors influencing NFR in hearing-impaired employees lags behind, although the outcome NFR has potential for early identification of hearing-impaired employees being at risk for occupational diseases, and may be a valuable tool for evaluating the effects of interventions aiming to prevent these problems. It is hypothesized that hearing loss, work characteristics, and personal characteristics influence both each other and NFR. Because earlier studies do not provide a framework on how these factors interfere, NFR may not be optimally understood in employees with hearing loss. The primary aim of this study is therefore to identify hearing-related, work-related, and personal factors influencing NFR in hearing-impaired employees. To examine if the influence of hearing-related, work-related, and personal factors on NFR differs from their influence on listening effort, which is a more commonly assessed construct when assessing the functional disability of hearing-impaired employees, the secondary aim is to identify factors influencing listening effort.

## Methods

### Design

We performed a single center study with an observational and cross-sectional design at the Amsterdam University Medical Center (Amsterdam UMC). Factors potentially influencing NFR were derived from hearing assessments performed at the hospital and a hearing survey that was completed at home. All variables were derived from patient files.

### Participants

We retrospectively identified patients referred to Amsterdam UMC’s ENT-Audiology department (location AMC) by their occupational physician. All patients were thus referred from occupational healthcare. Eligible patients visited the hospital between 2004 and 2019, were aged between 18 and 67, underwent pure tone and speech audiometry, and completed the hearing survey prior to their hospital visit. Patients were included regardless of the cause of their hearing loss. For patients with multiple referrals, the data were included belonging to the first referral with a completed questionnaire and hearing evaluation. To prevent bias, patients were excluded if they were referred to the hospital for a fitness for job assessment by their employer. The reason is that hearing loss complaints might be experienced or reported differently if continuation of the job depends on it. All patients received a letter with information about the study. Because of the retrospective study design, an opt-out procedure was performed.

**Fig. 1:**
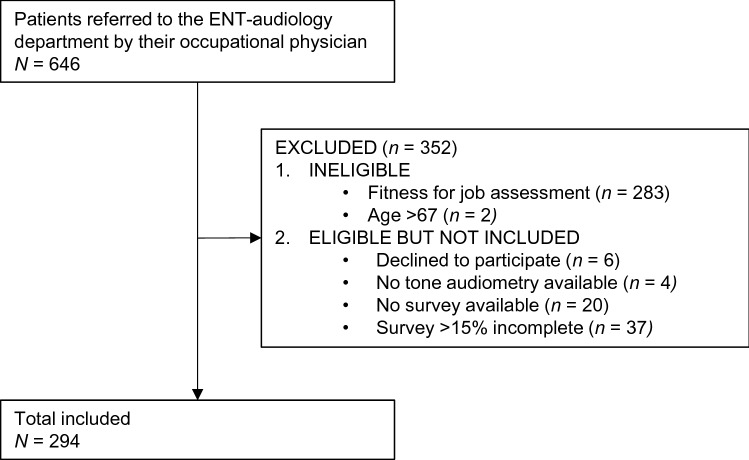
Flow chart

A total of 646 patients were identified of being referred to the ENT-Audiology department by their occupational physician (Fig. 1). Patients referred to the department to determine fitness for their job (*n* = 283) were not eligible for the study, as were patients older than 67 (*n* = 2). Further, patients were excluded that declined to participate (*n* = 6) or had an incomplete patient file, specifically missing tone audiometry (*n* = 4), missing survey (*n* = 20), or incomplete survey (*n* = 37).

### Data collection

The data were collected retrospectively by review of patient files. Outcomes of the hearing survey and the hearing assessments were entered into Castor, an electronic database (Castor EDC 2019) by the author and a research assistant. Data entry was checked in a sample of 50% of the cases. Information not available in patient files were noted as missing.

### Hearing assessment

The hearing assessments consisted of unaided pure tone and speech audiometry for all patients. At the ENT-Audiology Department, speech reception tests in noise are not routinely performed in all patients, but only if understanding speech in a noisy environment is important for job performance. Therefore, speech reception tests in noise were performed depending on the profession and the associated auditory demands.

#### Pure tone and speech audiometry

Pure tone and speech audiometry (ISO 8253–1, 1989) were performed in a sound-isolated booth using calibrated clinical audiometers (AC40 and Decos audioNigma) and TDH 39 headphones. According to the hospital protocol, pure tone thresholds for air and bone conduction were reported in decibel (dB) hearing level (HL) at frequencies 250 Hz, 500 Hz, 1000 Hz, 2000 Hz, 4000 Hz, and 8000 Hz, with adequate masking (if necessary). For a healthy, normally hearing individual, hearing thresholds up to 25 dB HL are regarded as normal (Martin and Champlin 2000). According to the American Academy of Otolaryngology, Binaural Hearing Impairment (BHI) was calculated from the mean of pure tone thresholds for air conduction at 1000 Hz, 2000 Hz, and 4000 Hz and a 5:1 weighting favoring the better ear (American Academy of Otolaryngology 1979). BHI provides a valid estimation of hearing disability that a person with a degree of hearing loss would experience (Dobie 2011). Speech audiometry was performed with the standard Dutch CVC word lists (Bosman and Smoorenburg 1995) and was used to calculate the percentage of maximum speech recognition for the better ear. It has been recommended to transform percentages of maximum speech recognition, to enhance the normality of the data for the statistical analysis (Sherbecoe and Studebaker 2004). Therefore, we transformed the percentages of maximum speech recognition using the rationalized arcsine formula (Sherbecoe and Studebaker 2004).

### Speech reception threshold test

Speech reception in continuous noise was assessed in a free field setting (S0N0) using everyday Dutch sentences developed by Plomp and Mimpen (1979) or the sentences developed by Versfeld et al. (2000). These sentence materials have been shown to result in similar test outcomes (Versfeld et al. 2000). The aim of performing a speech reception test in noise is to objectify the influence of the hearing loss on functional speech understanding at the workplace. Therefore, all patients were asked if they wear hearing aids at work. If they did, the critical Signal to Noise Ratio (SNR) was measured for aided conditions. If they did not, the SNR was measured for the unaided conditions. We have combined the SNR-outcomes of patients who performed the test with and without hearing aids. We will refer to these measurements as speech in noise tests performed in patient’s daily work situation. For a healthy, normally hearing individual, an SNR below − 4 can be expected (Versfeld et al. 2000).

### Survey

Prior to the hospital visit, patients completed an extensive hearing survey to investigate personal and environmental factors that potentially influence NFR. This survey consisted of three questionnaires and additional questions.

#### Experience and evaluation of work

Three scale scores were derived from the QEEW, a generic questionnaire on psychosocial workload and work stress (Van Veldhoven et al. 2002). The sum score of each QEEW scale can be converted to a scale score ranging from 0 to 100. A higher score represents a higher level of the working condition. First, the primary outcome was operationalized with the NFR-scale score that is composed of 11 items, such as “I find it hard to relax at the end of a working day”. An NFR-scale score higher than 54 indicates an increased risk for occupational and health problems (Broersen et al. 2004). Second, the score on the scale participation at work was included as a work-related factor. This scale consists of 8 items, such as “Can you participate in decisions about the nature of your work?”. Third, the score on the scale collegial support was included as a work-related factor. This scale consists of 9 items, such as “If necessary, can you ask your colleagues for help?” The QEEW has been shown to be reliable with good internal consistency and multiple studies have concluded good validity (Van Veldhoven et al. 2002, 2015). For example, the NFR scale has been shown to have good content-, construct-, and criterion-related validity in relation to work related health (Van Veldhoven and Sluiter 2009).

### The Amsterdam checklist for hearing and work

A 4-point response scale was used to inventory the occurrence of six hearing-related job activities, specifically detecting sounds, distinguishing sounds, communication in quiet, communication in noise, localizing sounds, and exposure to loud sounds. These questions on the occurrence of hearing-related job activities were merged into a value representing auditory demands at work by calculating a weighted sum score. Communication in quiet and distinguishing sounds are considered to be the easiest hearing activities and received a weighting of 1. Detecting and localizing sounds are considered to be of moderate difficulty and received a weighting of 2. Exposure to loud sounds and communication in noise are considered to be the most difficult and received a weighting of 3. This score can range between 0 and 48.

We did not only investigate the occurrence of the six hearing-related job activities, but also the effort they take. Since these six additional questions on the effort of hearing have good internal consistency (*α* = 0.81), we have calculated a sum score of these six items, further considered as self-perceived listening effort. This score can range between 0 and 18.

Other questions derived from the Amsterdam Checklist for Hearing and Work (ACHW) include the number of working hours a week (scale value) and fulfilling managerial tasks (dichotomous). All patients were asked whether they fulfilled managerial tasks, because managerial activities can be embedded in many professions and require specific skills that might appeal to hearing acuity, such as organizational and social skills (Whitley 1989).

### Communication Profile for the Hearing Impaired

To investigate the coping strategies, the CPHI was used. The CPHI aims to distinguish between adequate and inadequate coping behavior (Mokkink et al. 2009). It has been translated and validated for Dutch and contains two domains. The first domain regards communication strategies and contains 8 items for maladaptive behavior, 8 items for verbal strategies, and 7 items for nonverbal strategies. For example, items within the domain of communication strategies are “I avoid conversations, because of my hearing loss” (maladaptive behavior), “When I don’t understand what is being said, I ask for a repeat” (verbal strategies), and “I always try to watch a person’s face” (non-verbal strategies). The second domain regards personal adjustments and contains 6 items for self-acceptance, 8 items for acceptance of loss, and 15 items for stress and withdrawal. For example, items within the domain of personal adjustments are “I get mad at myself when I can’t understand others” (self-acceptance), “I can’t talk to people about hearing loss” (acceptance of loss), and “I get tense, because of my hearing loss” (stress and withdrawal). The CPHI scales are scored such that low scores are indicative of communicative of adjustment difficulties.

### Additional survey questions

Additional questions included the personal characteristics age, gender, educational level, and general health condition. For health condition, the response options were good, moderate, and bad. We used a dichotomous question to ask if the employees were feeling that something should change in their work situation. Two hearing-related characteristics were inventoried with a dichotomous question, specifically the presence of tinnitus, and the use of hearing aids.

### Variables in the analysis

A total of 17 factors potentially influencing NFR were explored for eligibility in the statistical model (Table 1). The same factors were explored for the secondary analysis. Hearing-related factors consisted of self-perceived listening effort, BHI, the maximum speech discrimination score, the critical Signal-to-Noise Ratio (SNR) measured in the speech-in-noise test, and the presence of tinnitus. Work-related factors consisted of work participation, collegial support, auditory demands at work, fulfilling managerial tasks, the number of working hours a week, and feeling that something should change at work. Personal factors consisted of the communication strategies and personal adjustments used, and general health condition. In addition, age, gender, and educational level were considered to be potential confounders.

**Table 1:** Factors hypothesized to influence on need for recovery

	Derived from	Operationalization
Binaural hearing impairment	Pure tone audiometry	Sum of pure tone thresholds at 1, 2, and 4 kHz with a 5:1 weighting favoring the better ear
Maximum discrimination	Speech audiometry	Percentage of maximum speech recognition for the better ear
SNR in continuous noise	Speech recognition test	SNR measured in the daily life situation at work (with or without hearing aids)
Presence of tinnitus	Survey4	Item score (dichotomous)
Work participation	Survey (QEEW)	Standardized scale score
Collegial support	Survey (QEEW)	Standardized scale score
Self-perceived listening effort	Survey (ACHW)	Sum score of 6 questions (4-point scale) on experienced listening effort during hearing-related job activities
Auditory work demands	Survey (ACHW)	Weighted sum score of 6 questions (4-point scale) on the occurrence of hearing-related job activities
Fulfilling managerial tasks	Survey (ACHW)	Item score (dichotomous)
Number of working hours	Survey (ACHW)	Item score (open question)
Feeling something should change	Survey	Item score (dichotomous)
Communication strategies	Survey (CPHI)	Standardized scale score consisting of maladaptive behavior, verbal strategies, and non-verbal strategies
Personal adjustments	Survey (CPHI)	Standardized scale score consisting of self-acceptance, acceptance of loss and stress, and withdrawal
Age	Survey	Item score (open question)
Gender	Survey	Item score (dichotomous)
General health condition	Survey	Item score (dichotomized)
Educational level	Survey	Item score (6 categories)

*SNR* Signal to Noise Ratio, *QEEW* Questionnaire on the Experience and Evaluation of Work; *ACHW* Amsterdam Checklist for Hearing and Work (ACHW), *CPHI* Communication Profile for the Hearing Impaired

### Statistical analysis

Distributions of all variables were examined. For continues variables, the means and standard deviations were calculated and histograms were used to check normality. For categorical variables, proportions were calculated. We drew a directed acyclic graph to reduce the required sample size and prevent power issues without missing factors related to the outcome measure and without missing factors required to reduce bias. This method aims to assist in the selection of appropriate variables for the regression analysis, as is recommended by Greenland et al. (1999). Afterwards, multiple linear regression was performed.

#### Directed acyclic graph

We visualized our hypothesized relationships between the factors and their association with the primary outcome NFR and secondary outcome self-perceived listening effort. To simplify the graph, we examined the correlations between the factors in the graph and removed all negligible associations, defined as correlation coefficients between − 0.3 and + 0.3 (Hinkle et al. 2003). Pearson correlation coefficients were used to examine the correlations between continuous variables, the Phi correlation coefficients for dichotomous variables, and the Bi-serial correlation coefficients to determine the correlation between a dichotomous and a continuous variable (Akoglu 2018; Kraemer 2014). Further simplification was accomplished by following the method of Shrier & Platt (2008), including removal of all factors that were not directly or indirectly related to neither the primary nor the secondary outcome.

### Multiple imputation

Multiple imputation was used to impute factors directly or indirectly related to the primary or secondary outcome (Pedersen et al. 2017). The number of imputations was 10, thus 10 imputed datasets were created. The imputation model consisted of all variables included in the conceptual model (Table 1).

### Linear regression analysis

Linear regression with a forward stepwise selection method (*α* = 0.05) was manually performed with all variables directly related to NFR. As a result of the strategy used to select factors for the analysis, the model was unadjusted for other factors. We checked for interaction effects with the use of hearing aids or not with all variables in the analysis, because the relationship between objective and subjective factors might be different for employees that wear hearing aids. If an interaction term was not found to be significant (*p* > 0.05), it was removed. Data organization and statistical analysis were performed using the Statistical Package for Social Sciences (SPSS) version 25.0 (Armonk New York USA). The critical value of significance was 0.05 for all statistical analyses.

## Results

### Participants

A total of 294 patients, mean age 56 (SD 8.9), were included the study (Table 2). Patients reported being in good health (60.2%), moderate/poor health (39.1%), and poor health (0.7%). Since 0.7% of the cases used the third category, this question was dichotomized for the statistical analysis. The mean BHI was 41.3 dB HL (SD 20.76). For the maximum speech discrimination score in quiet, the median was 100% (range 15%–100%). The mean critical SNR was -2 dB (SD 4.4). Hearing aids were used by 58.5% of the patients.

**Table 2:** Characteristics of the included participants (*N* = 294)

	%	Mean (SD)	Min; max	Missing *n*
Age		50.9 (8.9)	19; 65	0
Gender (% male)	58.6			0
General health condition				2
Good	60.2			
Moderate/poor	39.1			
Degree of hearing loss (weighted)				0
Normal hearing (< 15 dB HL)	22.9			
Mild (25–40 dB HL)	28.0			
Moderate (40–60 dB HL)	31.7			
Severe (60–80 dB HL)	11.3			
Profound (> 80 dB HL)	6.1			
Binaural hearing impairment^a^		41.3 (20.8)	3.8; 110.8	0
Maximum discrimination		94.5 (12.9)	15; 100	1
SNR in continuous noise		− 2.2 (4.4)	− 9; 14.6	158
Presence of tinnitus (% yes)	63.9			4
Hearing aids (% yes)	57.5			3
Educational level				5
Primary/lower vocational	7.6			
General intermediate	7.6			
Intermediate vocational	22.5			
General secondary	10.0			
Higher vocational	36.0			
University	16.3			
Profession				1
Teacher	26.6			
Administrative	19.5			
Doctor/nurse	10.2			
Manager	9.2			
Coach/social worker	5.5			
Construction worker	4.4			
Police officer/fireman	3.4			
Other	21.2			
Number of working hours		33.6 (8.7)	16; 48	1
Fulfilling managerial tasks (% yes)	88.4			21
Need for recovery (range 0–100)		54.9 (34.1)	0; 100	12
Work participation (range 0–100)		49.0 (22.8)	0; 95.83	11
Collegial support (range 0–100)		20.8 (13.2)	0; 55.56	14
Self-perceived listening effort (range 0–18)		10.3 (4.1)	0; 18	20
Auditory demands at work (range 0–48)		30.6 (6.1)	16; 48	10
Feeling something should change (% yes)	45.2			24
Communication strategies (range 23–115)		79.8 (15.8)	0; 115	7
Personal adjustments (range 29–145)		97.3 (26.2)	0; 145	7

*SNR* Signal to Noise ratio

^a^Binaural hearing impairment is defined as the mean of the pure tone averages of the left and right ear with a 5:1 weighting favoring the better ear

All educational levels were represented. The most common professions were teacher (26.6%), administrative job (19.4%), doctor/nurse (10.2%), and managerial jobs (9.2%). Many patients with and without managerial jobs reported to fulfil managerial tasks (88.4%). The mean number of working hours per week was 33.6 (SD 8.7). The mean score for NFR was 54.94 (SD 34.12). In 55.8% of the participants, the NFR score was above 54, indicating an increased risk at occupational and health problems (Broersen et al. 2004). The mean self-perceived listening effort was 10.28 (SD 4.05). The CPHI resulted in a mean score of 79.79 (SD 15.84) for communication strategies and 97.31 (SD 26.23) for personal adjustments. A normal distribution was confirmed for all variables, except for the percentage of maximum speech recognition. Even after application of the rationalized arcsine transformation (Sherbecoe and Studebaker 2004), the variable remained skewed to the right. This variable was therefore not used in the analysis.

### Directed acyclic graph

**Fig. 2:**
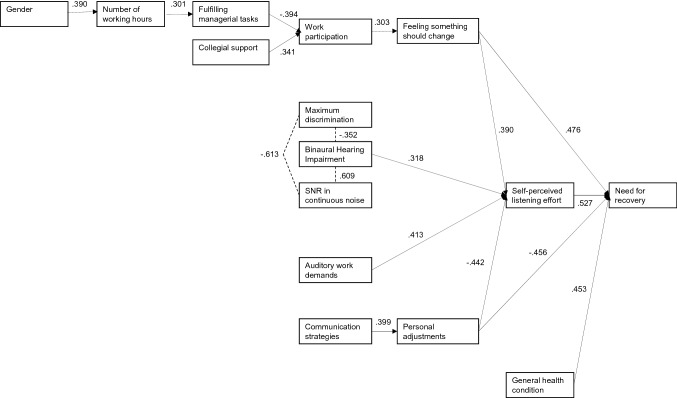
Directed acyclic graph: a visualization of our hypothesized relationships between the factors and need for recovery that was simplified by removing all correlations between − 0.3 and + 0.3, followed by simplification according to the method of Shrier and Platt (2008). *SNR* Signal-to-Noise Ratio

The presence of tinnitus, age, and the educational level were not directly or indirectly associated with NFR and self-perceived listening effort. Consequently, these factors were not included in the directed acyclic graph. Figure 2 shows the directed acyclic graph that was constructed. Four variables directly influenced the primary outcome NFR, specifically feeling that something should change at work (*r* = 0.476), self-perceived listening effort (*r* = 0.527), personal adaptations (*r* = − 0.456), and general health condition (*r* = 0.453). Two of these variables did also directly influence the secondary outcome measure self-perceived listening effort, respectively, feeling that something should change at work (*r* = 0.390), and personal adaptations (*r* = − 0.442). Self-perceived listening effort was also directly influenced by BHI (*r* = 0.318) and auditory work demands (*r* = 0.413). The hearing assessment outcomes did not significantly correlate with NFR, including BHI (*r* = 0.096, *p* = 0.109), maximum discrimination score (*r* = 0.010, *p* = 0.873), and SNR (*r* = 0.060, *p* = 0.492). All correlations between the hypothesized factors and the primary and secondary outcome are shown in Table 3.

**Table 3:** Correlations between the hypothesized factors and the primary outcome need for recovery and the secondary outcome self-perceived listening effort

	Need for recovery	Self-perceived listening effort
Binaural hearing impairment	0.099	0.318
Maximum discrimination	− 0.024	− 0.167
SNR in continuous noise	0.060	0.203
Presence of tinnitus	0.102	0.094
Work participation	0.154	0.006
Collegial support	0.198	0.130
Self-perceived listening effort	0.527	–
Auditory work demands	0.226	0.413
Fulfilling managerial tasks	– 0.050	0.109
Number of working hours	– 0.152	− 0.071
Feeling something should change	0.476	0.390
Communication strategies	0.032	0.197
Personal adjustments	– 0.456	− 0.442
Age	− 0.018	0.133
Gender	− 0.186	− 0.133
General health condition	0.453	0.289
Educational level	0.108	0.124

*SNR* Signal to Noise Ratio

### Multiple regression analysis

The results of the primary regression analysis (Table 4) indicated the four predictors explained 46.1% of the variance (*p* < 0.001) of the NFR. Feeling that something should change at work, self-perceived listening effort, the personal adaptations scale score, and having a moderate/poor general health condition were significantly related to higher NFR. In the secondary regression analysis (Table 5), the four predictors explained 43.1% of the variance (*p* < 0.01) of the self-perceived listening effort. Feeling that something should change at work, BHI, auditory work demands, and the personal adaptations scale score were significantly related to self-perceived listening effort. In both analyses, there were no significant interaction effects.

**Table 4:** Results of multiple linear regression analysis of factors associated with *need for recovery*

	Variable	*B*	95% CI	*p*
Complete case analysis *R*^2^ = 0.495	Constant	32.03	9.12; 54.93	.006
	Feeling something should change*	19.01	12.04; 25.97	< .001
	Self-perceived listening effort	1.84	0.88; 2.81	< .001
	Personal adaptations	− 0.34	− 0.49; − 0.19	< .001
	General health condition**	20.06	13.18; 26.94	< .001
Pooled analysis after imputation *R*^2^ = 0 .461	Constant	31.78	8.70; 54.86	.010
	Feeling something should change*	17.88	10.48; 25.29	< .001
	Self-perceived listening effort	1.93	0.97; 2.88	< .001
	Personal adaptations	− 0.31	− 0.45; − 0.16	< .001
	General health condition**	17.99	11.44; 24.53	< .001

*Reference category = not feeling that something should change in the work situation

**Reference category = being in good health

**Table 5:** Results of multiple linear regression analysis of factors associated with *self-perceived listening effort*

	Variable	*B*	95% CI	*p*
Complete case analysis *R*^2^ = .0408	Constant	6.78	3.77; 9.80	< 0.001
	Feeling something should change*	1.96	1.12; 2.80	< 0.001
	Binaural hearing loss	0.01	0.01; 0.02	< 0.001
	Auditory work demands	0.19	0.12; 0.26	< 0.001
	Personal adaptations	− 0.05	− 0.06; − 0.03	< 0.001
Pooled analysis after imputation *R*^2^ = 0.431	Constant	6.10	3.35; 8.84	< 0.001
	Feeling something should change*	1.82	1.03; 2.61	< 0.001
	Binaural hearing impairment	0.01	0.01; 0.02	< 0.001
	Auditory work demands	0.20	0.14; 0.26	< 0.001
	Personal adaptations	− 0.05	− 0.06; − 0.03	< 0.001

*Reference category = not feeling that something should change in the work situation

## Discussion

The aim of this study was to identify factors influencing NFR and listening effort in hearing-impaired employees. Four factors were shown to directly influence NFR and four factors were shown to directly influence self-perceived listening effort.

In line with the literature arguing the theoretical assumption that increased listening effort may cause a sense of mental fatigue (McGarrigle et al. 2014), self-perceived listening effort was found to be the factor with the highest association with NFR (*r* = 0.527) in the correlation analysis. In contrast, no significant associations were observed between the hearing test outcomes and NFR, including BHI, maximum discrimination score, and the critical SNR. While tinnitus has earlier been shown to be associated with NFR (Juul Jensen et al. 2018), we did not find a significant association in this study. This may be explained by the dichotomous question that we used that did not allow for differentiating in degree of tinnitus. Also, because we used routinely obtained healthcare data, we may have missed hearing-related factors, such as hyperacusis. For concepts related to NFR, mixed results are presented for hearing loss (Hornsby and Kipp 2016; Svinndal et al. 2018). Tone audiometry was not significantly related to fatigue and vigor (Hornsby and Kipp 2016), but patients with more severe hearing loss reported lower workability and higher degrees of fatigue (Svinndal et al. 2018).

The lack of a significant association between SNR and NFR in the correlation analysis contrasts the results of an earlier study that found poorer SNR to be associated with higher NFR (Nachtegaal et al. 2009). In this earlier study, SNR was derived from an adaptive digits-in-noise test performed over the internet and the subjects completed the test without hearing aids. In our study, routinely healthcare data were used, having the advantage that all hearing tests were performed in a standardized audio cabins, but with the disadvantage that SNR data were missing in 158 patients (54%). Performing the speech reception test in noise is not obligatory in standard care. The choice to perform the speech reception test is determined by a patient’s profession and associated auditory demands. Therefore, the missing SNR data are not missing at random, and the presence of confounding cannot be ruled out. Another explanation might be that we derived SNR’s with and without hearing aids, to resemble patients’ daily life work situation. Although we expected the SNR scores to be more strongly associated with NFR, this choice may have masked an existing association.

Since BHI correlates with self-perceived listening effort, but not with NFR, we presume that the degree of hearing loss is not the underlying factor explaining the moderate correlation between listening effort and NFR. In the directed acyclic graph, two factors show moderate correlations with both self-perceived listening effort and NFR, specifically: feeling that something should change at work and personal adaptations. Apparently, the way employees perceive their hearing difficulties and how they cope with their hearing loss influence their listening effort and the fatigue experienced after a day of work. Likewise, subjective measures of perceived hearing difficulties were found to be strongly associated with fatigue and vigor, whereas there was no significant association with degree of hearing loss (Hornsby and Kipp 2016). It would be interesting to compare our findings of self-reported listening effort with other measures of listening effort, such as measuring reaction time or pupil responses during speech reception tasks in noise (McGarrigle et al. 2014).

In line with de Vries et al. (2015) and Machin and Hoare (2008), we found a significant correlation between coping behavior and NFR. Specifically, we have explored two variables for coping behavior distinguishing the communication strategies that were used and the personal adaptations that were made. Although these scores showed a moderate correlation between themselves (*r* = 0.399), the personal adaptation score was directly related to self-perceived listening effort and NFR, but the communication strategy score was not. Other studies report an association between communication strategies used and NFR (de Vries et al. 2015; Machin and Hoare 2008). Having a passive reaction coping style explained 26% of the variance in NFR in employees with major depression in remission (de Vries et al. 2015). In a population of bus drivers, maladaptive driver coping behaviors were shown to be associated with NFR (Machin and Hoare 2008). To our knowledge, previous studies have not focused on the association between NFR and personal adaptations, including self-acceptance, acceptance of loss, and stress and withdrawal. A qualitative study reported that self-acceptance facilitates work ability (Detaille et al. 2003). Distinguishing communication strategies and personal adaptations would be of interest in future studies with hearing-impaired employees to gain further understanding of the influence of coping behavior on NFR.

In addition to explore the influence of coping behavior, several other questions were included to assess the influence of personal factors. We observed that the factor feeling that something should change at work was moderately associated with NFR, as well as with self-perceived listening effort. The question “Do you feel something should change in your work situation” may grasp a feeling of frustration at the workplace, that was earlier associated with NFR in seafarers (Bridger et al. 2010). Feeling frustration at the workplace might be associated with higher need for recovery. This finding must; however, be interpreted with caution, because we used a single question, rather than a validated questionnaire. The question may also reflect other constructs, such as the awareness or acceptance of functional hearing difficulties at the workplace. Although a firm conclusion can thus not yet been drawn, this finding underlines the importance to measure employees’ frustration level in future research concerning NFR using a validated questionnaire.

In line with the previous studies (Gommans et al. 2015; van der Starre et al. 2013), general health condition was found to be significantly associated with NFR, independently from the other factors. Age, gender, and educational level were considered to be potential confounders, but the correlation analysis showed that these factors were neither significantly associated with NFR, nor with hearing related or personal factors. A similar independent position was found for the factor auditory demands. This factor was moderately associated with self-perceived listening effort. In contrast to the literature describing that auditory demands are significantly related to hearing handicap and sick leave (Kramer et al. 1998, 2006), we did not find a significant association between auditory demands at work and NFR. This may suggest that although high auditory demands increase the listening effort, the degree of feeling fatigued after work depends on other factors. The use of personal adaptations or being in good health may be protective for developing occupational problems. Future research is required to further assess these mechanisms.

Other work characteristics did neither influence NFR nor self-perceived listening effort. First, we expected a positive association between the number of working hours and NFR (Jansen et al. 2002; Verdonk et al. 2010), but this was not the case. The lack of association could be explained if patients with high NFR had chosen to work fewer hours to prevent health problems. Since this study uses health administrative data, we cannot confirm this hypothesis. The directed acyclic graph showed that men had a higher number of working hours than women, which is a typical finding for the Dutch working population (Gjerdingen et al. 2001). Employees that reported a higher number of working hours, more often reported fulfilling managerial tasks and those fulfilling managerial tasks reported being more able to participate in work decisions. Second, in contrast to what was observed earlier (van Veldhoven and Broersen 2003), work participation was not associated with NFR. In other words, the feeling of job control did not directly influence NFR. Literature presents mixed results on the association between job control and NFR (Kraaijeveld et al. 2014; Sonnentag and Zijlstra 2006; Van der Hulst et al. 2006). Third, receiving collegial support did neither influence the feeling that something should change at work, nor NFR as was earlier reported (Kraaijeveld et al. 2014). This might be explained by the small variance in collegial support reported by our population. Only a few employees reported having problems in their relationship with colleagues.

We have derived work-related factors from the QEEW and ACHW, because these questionnaires are routinely performed in the ENT-Audiology clinic. Therefore, we may have missed other work-related factors that influence NFR in hearing-impaired employees, such as job control, job demand, and social support. The included scale score of collegial support does not reflect all aspects of the construct social support, since this construct also refers to helpful social interactions from supervisors (Nachtegaal et al. 2009). For future research, we recommend to include the Job Content Questionnaire when measuring psychosocial work characteristics (Karasek et al. 1998).

Some study limitations should be noted. First, the retrospective character of the study implicates a risk for measurement bias. For example, the hearing tests were performed by multiple clinicians following clinical protocols, rather than a research protocol, which may have caused differences in measurement settings. Despite this limitation, the four identified factors accounted for 46.1% of the variance in NFR and 43.1% of self-perceived listening effort. Second, the cross-sectional design is a limitation of this study, since it does not allow drawing conclusions about causality. Constructing a directed acyclic graph allowed for visualization of the relationship of a broad spectrum of factors influencing NFR. Since the evidence on factors influencing NFR in hearing-impaired employees was limited, this explorative method is considered to be appropriate. A prospective study is needed to verify and validate the findings of this study. To gain further understanding in the difficulties of hearing-impaired employees and the efficacy of intervention strategies that aim to reduce these difficulties, future clinical trials are recommended to assess the efficacy of audiological, speech therapeutic, and social interventions on both self-perceived listening effort and NFR.

### Concluding remarks

This study provides a framework of factors associated with NFR in hearing-impaired employees, contributing to the understanding of occupational problems in this population. The results suggest that the way employees perceive their hearing loss and how they cope with it directly influence NFR, rather than their measured degree of hearing loss. Further, when assessing or evaluating NFR, an employees’ general health condition should be considered. These findings are relevant for clinicians and occupational physicians that perform diagnostics or intervention strategies for hearing-impaired employees. Also, the results may contribute to gain understanding in the working mechanisms of interventions that aim to prevent or cure occupational diseases in employees with hearing loss.
